# Nutrients exported from upland stream water enlarge perennial biomass crops

**DOI:** 10.1038/s41598-021-81191-x

**Published:** 2021-01-25

**Authors:** Masaaki Chiwa, Yasuhiro Utsumi, Naoaki Tashiro, Yuko Yasuda, Ken’ichi Shinozuka, Yang Ru, Nao Nagano, Shusuke Murata, Takuma Nakamura, Kohei Yamauchi, Yuji Kabemura, Tatsuro Ando, Hiroshi Sawamura

**Affiliations:** 1 Kyushu University Forest, Kyushu University, 394 Tsubakuro, Sasaguri, Fukuoka, 811-2415 Japan; 2 Forestry and Forest Products Research Institute, 1 Matsunosato, Tsukuba, Ibaraki 305-8687 Japan; 3 Fukuoka Institute of Technology, 3-30-1 Wajiro-higashi, Higashi-ku, Fukuoka, 811-0295 Japan; 4 Ashoro Museum of Paleontology, 1-29-25 Konan, Ashoro, Hokkaido 089-3727 Japan

**Keywords:** Biogeochemistry, Ecology, Biogeochemistry, Ecology, Environmental sciences

## Abstract

Rawanbuki, a variety of Japanese butterbur (*Petasites japonicus* subsp. *giganteus*), grow naturally along the Rawan River, Hokkaido, northern Japan. Most plants reach 2–3 m in height and 10 cm in diameter in 2 months and are much larger than those grown along other rivers. We examined the hypothesis that nutrients exported from upland streams enhance the growth of the Rawanbuki. Nutrient concentrations, including nitrogen, phosphorus, and base cations, in the Rawan River were much higher than those in rivers of adjacent watersheds. High nutrient concentrations and moisture contents were found in soil along the Rawan River and a significant relationship was found between physicochemical soil conditions and aboveground biomass of butterburs. This indicates that extremely large Rawanbuki plants could be caused by these high nutrient concentrations and moisture contents in the soils. A manipulation experiment showed that fertilization simulated the growth environment along the Rawan River and enhanced the stem height and stem diameter of butterburs. This study concluded that the extremely large butterburs are caused by a large amount of nutrients exported from upland areas. These results are the first demonstration of the role of stream water nutrients in enlarging agricultural crops.

## Introduction

Rawanbuki, a variety of the Japanese butterbur Akitabuki (*Petasites japonicus* subsp. *giganteus*), is grown naturally along the Rawan and Moashoro Rivers, Hokkaido, northern Japan (Fig. S1). This species is registered as Hokkaido Heritage, and the young stem is used as a vegetable. Akitabuki is common in wet and sunny places but also tends to form thickets on the banks of rivers. Most Rawanbuki plants reach 2–3 m in height and 10 cm in diameter in 2 months, and these are much larger than those grown along other nearby rivers, including the Toshibetsu River (Fig. S1). However, the reason that Rawanbuki plants are extremely large remains unknown.

Imazu^1^ showed that *Petasites japonicus* from northern to southern Japan including cool to warm temperate zones had a wide range of variation in morphological characters when they were grown under the same conditions, but did not found any definite geographical differentiation of these characters. In addition, Imazu and Fujishita^2^ found that Akitabuki is a diploid, whereas *Petasites japonicus* grown in southern Japan is either triploid or diploid. These results imply that the enlargement effect on Akitabuki would not be genetic because ploidy has a positive effect on plant growth in general^3,4^.

Preliminary experiments showed that nutrients including nitrogen, phosphorus, and minerals in the Rawan and Moashoro Rivers are abundant compared to those in other neighboring streams, including the Toshibetsu and Ashoro Rivers. The concentrations of nitrate (NO_3_^−^), soluble reactive phosphorus (SRP), sodium (Na^+^), potassium (K^+^), magnesium (Mg^2+^), and calcium (Ca^2+^) in the Rawan and Moashoro Rivers were approximately 6–9, 2–3, 7–8, 7, 8–11, and 3–6 times higher than the Toshibetsu and Ashoro Rivers, respectively (Fig. S2). Therefore, we hypothesize that nutrients exported from upland areas stimulate the growth rates of Rawanbuki, resulting in them being extremely large.

Few studies have focused on the role of stream water nutrients in enlarging agricultural crops, whereas many studies have reported the effects of agricultural activities as pollutant sources on stream water quality. Bioremediation technologies are considered emerging and sustainable methods for the remediation of environmental pollutants^5^. Stream water nutrients may enlarge agricultural crops, which may provide a strategy for climate change mitigation^6^ because larger crops can take up more carbon dioxide.

To test this hypothesis that nutrients exported from upland areas stimulate the growth rates of Rawanbuki, three experiments were conducted: (1) a detailed analysis of the stream water quality along the Rawan and Moashoro Rivers, (2) an evaluation of the growth and environmental conditions of Rawanbuki, including soil nutrients, and (3) a manipulation experiment to test fertilization effects on the growth of butterburs.

## Results and discussion

### Stream water quality along the Rawan River

**Figure 1:**
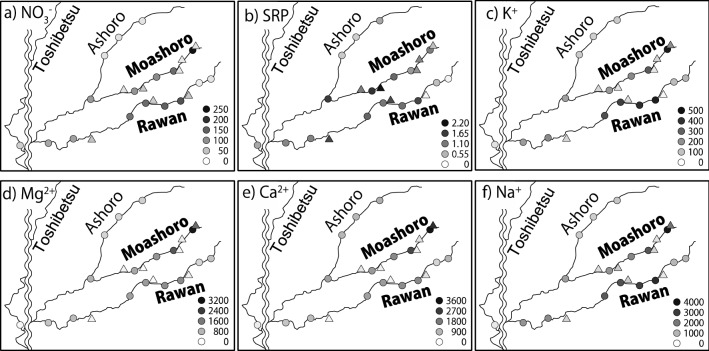
Spatial variations in concentrations of (**a**) NO_3_^−^, (**b**) soluble reactive phosphorus (SRP), (**c**) K^+^, (**d**) Mg^2+^, (**e**) Ca^2+^, and (**f**) Na^+^ in the main streams (circles) and tributaries (triangle) to the main streams of the Rawan, Moashoro, Ashoro, and Toshibetsu Rivers. The map was generated from Google Maps.

Solute concentrations, including NO_3_^−^ (Fig. 1a), SRP (Fig. 1b), and minerals (Fig. 1c–f), in the Rawan and Moashoro Rivers were much higher than those in the neighboring rivers, including the Toshibetsu and Ashoro Rivers. Nutrient concentrations including NO_3_^−^, SRP, and minerals in the Rawan and Moashoro Rivers were comparable to the maximum values of solute concentrations in stream water compiled by Kobayashi^7^, who investigated the water quality of 225 streams throughout Japan (Table S1).

The underlying geology in the Rawan and Moashoro Rivers is very different from that in the Toshibetsu and Ashoro Rivers (Fig. S3), and this geology can characterize the stream water quality in the Rawan River. Specifically, the high percentage of Holocene nonalkaline mafic volcanic rocks in Rawan A and Moashoro A (Fig. S3) could increase the NO_3_^−^, SRP, and mineral concentrations of stream water in the Rawan and Moashoro Rivers.

Stream water NO_3_^−^ concentrations in the Rawan and Moashoro Rivers were more than 100 μmol L^−1^, which was much higher than the average concentration of NO_3_^−^ (25 μmol L^−1^)^8^ in the water of 34 streams in forests in Japan and comparable with that reported in streams in urban/suburban forests^9–11^. Higher NO_3_ concentrations in stream water are generally caused by agricultural activity^12^. However, our results showed that the NO_3_^−^ concentration was also high in stream water from upland forested areas. Elevated atmospheric N deposition is also generally responsible for the high concentration of NO_3_^−^ in stream water from forested watersheds^13^. However, atmospheric N deposition in this region (4.4 kg N ha^−1^ year^−1^)^14^ is not high enough to increase the NO_3_^−^ concentration in stream water. Although the reason remains unclear, weathering of N from bedrock^15,16^ could be a possible source for relatively high NO_3_^−^ concentrations in stream water in this study.

SRP concentrations in the Rawan and Moashoro Rivers were also high (Fig. 1b). As was the case with NO_3_^−^, agricultural activity is responsible for the higher concentration of SRP in stream water^12^. However, the SRP concentration was also high in stream water from upland forested areas in this study. Wakamatsu et al.^17^ reported that the median concentration of SRP in the water collected from 1244 streams throughout Japan was 0.0066 mg L^−1^ (0.21 μmol L^−1^), with a maximum concentration of 0.116 mg L^−1^ (3.7 μmol L^−1^). In their study, the SRP concentration in stream water was higher (> 0.01 mg L^−1^) around Mt. Meakan than in other locations, and that area is included in this study area (Fig. S1a). Geology is an important factor affecting SRP concentration in stream water^17,18^. Phosphorus in rocks is contained almost entirely in apatite group minerals and is high in common iron-rich rocks^19^. The SRP concentration in stream water is considered to be affected by rock weathering^17^. Therefore, bedrock geology around the Rawan and Moashoro Rivers (Fig. S3) could cause high concentrations of SRP in the Rawan River originating from upland forests.

The concentrations of base cations, including magnesium (Mg^2+^), calcium (Ca^2+^), potassium (K^+^), and sodium (Na^+^), in the Rawan and Moashoro Rivers were 10–20 times higher than those in the Toshibetsu River (Fig. 1c–f). These higher concentrations of base cations in the Rawan and Moashoro Rivers than in the Toshibetsu River could also be due to geology. Overall, stream water quality, including nitrate, SRP, and minerals, along the Rawan and Moashoro Rivers could be characterized by bedrock geology that is very different from that in the Toshibetsu and Ashoro Rivers (Fig. S3).

### Growth and conditions of Rawanbuki along the Rawan River

**Figure 2:**
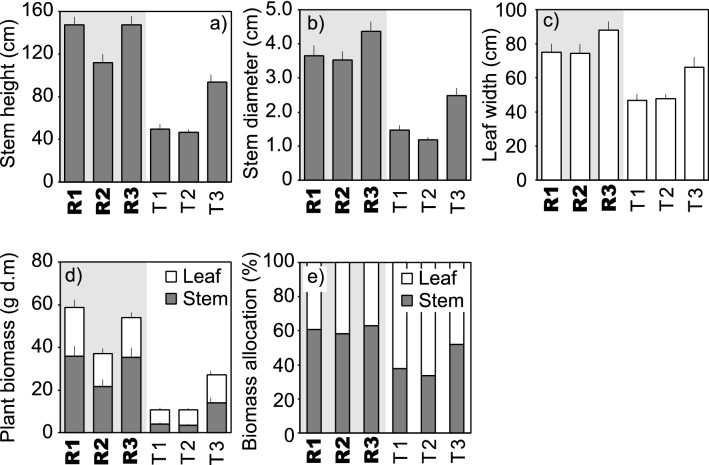
(**a**) Stem height, (**b**) stem diameter, (**c**) leaf width, (**d**) plant biomass, and (**e**) biomass allocation of butterburs grown along the Rawan (R1, R2, and R3) and Toshibetsu (T1, T2, and T3) Rivers. Bars represent standard errors [*n* = 37 (R1), 21 (R2), 25 (R3), 21 (EA), 35 (EB), and 22 (EC)]. The difference between the Rawan and Toshibetsu Rivers was significant for stem height, stem diameter, leaf width, plant biomass, and stem ratio of biomass. See Fig. S1 for the locations of R1-3 and T1-3.

Comparisons of the size (Fig. 2a–c) and weight (Fig. 2d) of butterburs grown between the Rawan (R1, R2, and R3) and the Toshibetsu (T1, T2, and T3) Rivers showed that the Rawanbuki plants grown along the Rawan River is much larger than those along the Toshibetsu River. Rawanbuki plants were tall (more than 1 m in height, Fig. 2a) and thick (more than 3 cm diameter, Fig. 2b), and their leaves were large (more than 70 cm width, Fig. 2c). The substantial biomass of Rawanbuki plants (Fig. 2d) was allocated most to stems (Fig. 2e).

**Figure 3:**
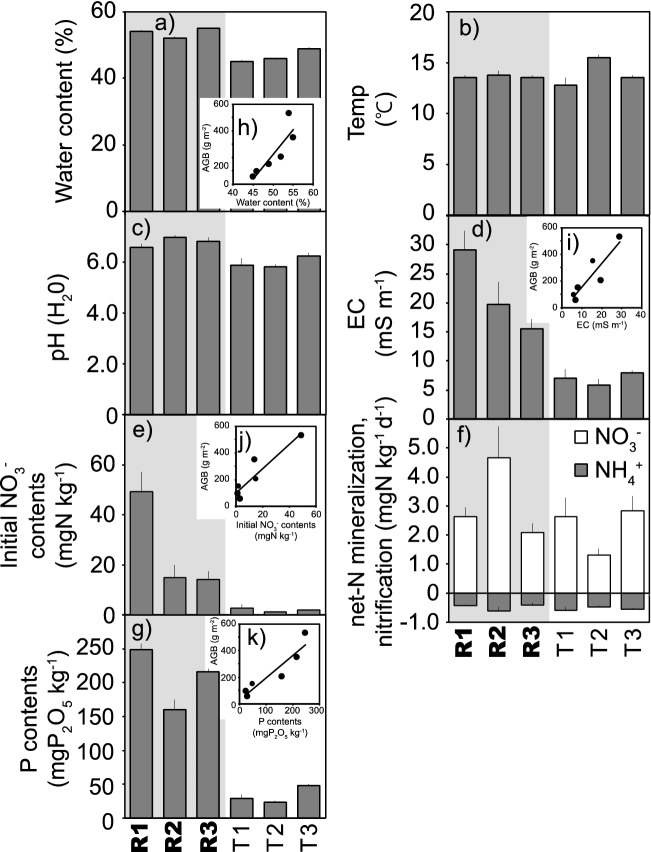
Soil physicochemical conditions for (**a**) soil water content, (**b**) temperature, (**c**) pH (H_2_O), (**d**) electric conductivity (EC), (**e**) initial NO_3_^−^ contents, (**f**) net N mineralization and nitrification, and (**g**) initial available P contents along the Rawan (R1, R2, and R3) and Toshibetsu (T1, T2, and T3) Rivers and the relationship with plant biomass for (**h**) soil water content, (**i**) EC, (**j**) initial NO_3_^−^ contents, and (**k**) initial available P contents. The figure without a relationship with plant biomass indicates no significant relationship with plant biomass. The difference between the Rawan and Toshibetsu Rivers was significant for soil water content, pH (H_2_O), EC, initial NO_3_^−^ contents, and initial available P contents. See Fig. S1 for the locations of R1-3 and T1-3.

The high level of nutrients and water along the Rawan River (Fig. 3a–g, Table S2) and the significant relationship between physicochemical soil conditions and aboveground biomass of butterburs (Fig. 3h–k) indicate that extremely large Rawanbuki plants could be caused by these high levels of nutrients and water in the soils. The soil water content (Fig. 3a), electric conductivity (EC) (Fig. 3d), initial NO_3_^−^ content (Fig. 3e), and P content (Fig. 3g) were higher along the Rawan River (R1, R2, and R3) than along the Toshibetsu River (T1, T2, and T3). Thus, there were significant relationships between aboveground biomass and soil water content (Fig. 3h), soil EC (Fig. 3i), initial NO_3_^−^ content (Fig. 3j), and P content (Fig. 3k).

Nutrient-rich conditions in the Rawan River likely result from upland stream water inflow. This scenario is supported by the significant relationships between soil (Table S2) and stream water (Table S3) in terms of pH (Fig. S4a), EC (Fig. S4b), P (Fig. S4c), and NO_3_^−^ (Fig. S4d). The significant relationship between aboveground biomass and initial soil NO_3_^−^ content (Fig. 3e) and the lack of a significant relationship between biomass and soil net-N mineralization support the concept that nutrients supplied from upland areas via stream water enhance soil nutrient conditions, resulting in extremely large Rawanbuki plants.

Many studies have reported the effects of agricultural activities as pollutant sources on stream water quality^12,20^. In contrast, few studies have demonstrated the effects of stream water quality on agricultural products. Aquatic plants play an important role in improving stream water quality, such as through bioremediation in urban rivers^21^. In addition, stream water quality can affect the growth of algae^21^ and microbes^22^. Therefore, plants and microbes utilize nutrients supplied from stream water. To our knowledge, our results are the first demonstration of the role of nutrients exported from upland stream water in the enlargement of agricultural crops.

Despite the fact that soil nutrients are abundant along the Rawan River, tissue N (Fig. S5a,c) and P concentrations (Fig. S5b,d) of Rawanbuki plants (R1, R2, and R3) did not differ significantly from those of Rawanbuki plants along the Toshibetsu River (T1, T2, and T3). This scenario could have been caused by Rawanbuki plants decreasing the abundance of nutrients as the N and P content of plant tissue is diluted by the accumulation of carbohydrates, and these plants had greater biomass along the Rawan River than along the Toshibetsu River^23^. Significant relationships between soil nutrients and aboveground nutrient contents of N (Fig. S6a) and P (Fig. S6b) indicate that soil nutrients were taken up by the plants in proportion to the soil nutrient contents.

### Manipulation experiment simulating soil nutrient conditions along the Rawan River

**Figure 4:**
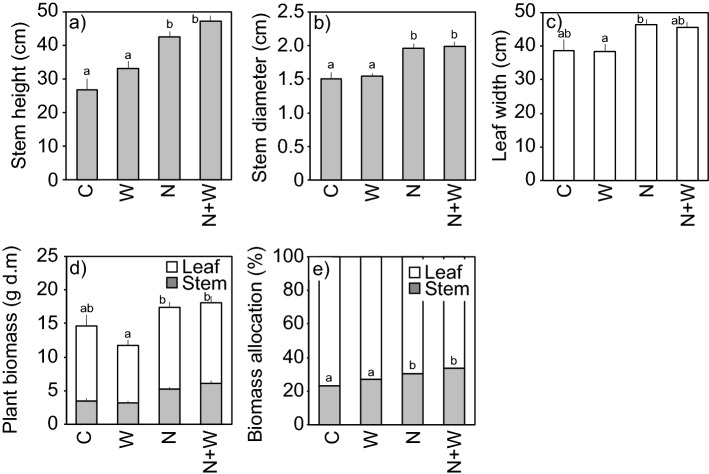
(**a**) Stem height, (**b**) stem diameter, (**c**) leaf width, (**d**) plant biomass, and (**e**) biomass allocation of butterburs grown under the conditions of the control (C), addition of water (W), addition of nutrients (N), and addition of nutrients and water (N + W). Bars represent standard errors [*n* = 8 (C), 9 (W), 8 (N), 8 (N + W)]. Different letters indicate significant differences at *P* < 0.05 (Tukey’s honest significant difference test) among the treatments.

Our manipulation experiment confirms the hypothesis that additional nutrients enhance the size of butterburs. Stem height (Fig. 4a) and stem diameter (Fig. 4b) were significantly greater for the nutrient-addition plots (N and N + W) than for the control (C) and water-addition plot (W). Leaf width (Fig. 4c) was also greater for nutrient-addition plots (N and N + W) than for the other plots, though not significantly. Although the difference in the aboveground biomass of the individuals, such as that of leaves and stems, between the nutrient-addition plots (N and N + W) and the control (C) and the water additional plot (W) was small (Fig. 4d), the difference in biomass was evident for the area-based plot (Fig. S7), which was calculated by dividing the sum of the individual aboveground biomass value by the plot area. Our results were consistent with those of manipulation studies showing the effect of nitrogen on the productivity of ryegrass^24^, perennial shrubs and grasses^25^, annual grasses^26^ and perennial biomass crops^27^. It has also been shown that nutrient addition, including nitrogen, phosphorus, and potassium, increases primary production of rhizomatous plants^28^, various vascular species^29^, and aboveground biomass in mesotrophic fens^30^.

The effect of water supply (plot W) was smaller than that of nutrient supply (plot N) (Fig. 4, Fig. S7). During the irrigation process, the soil water potential fluctuated from − 0.09 to − 15.9 kPa and the average was − 10.5 kPa. These values are higher than the soil water stress thresholds for many crops^31^. These results indicate that nutrients are a main cause of the large Rawanbuki. In comparison to other sizes of butterburs (C and W), large butterburs (N and N + W) allocated to more stems (Fig. 4e), which was consistent with the field measurements where large Rawanbuki allocated more stems (Fig. 2e).

No significant difference in the area-based maximum net carbon dioxide (CO_2_) assimilation rates (*A*_*max*_) existed among the treatments (Fig. S8), indicating that butterburs increased photosynthate by increasing leaf area instead of increasing the capacity of area-based photosynthesis. The small difference in the leaf nitrogen concentration of the butterburs among the treatment plots (Fig. S9) may support the small difference in area-based *A*_*max*_ among the treatments. In addition, the small difference in the leaf nitrogen concentrations of the butterburs was consistent with the results of the field measurements, where no significant difference was found for the leaf nitrogen concentrations of the butterburs between the Rawan and Toshibetsu Rivers (Fig. S5a).

## Methods

### Study site

The Rawan and Moashoro River basins, the natural habitat of large Rawanbuki, are located in the eastern part of Hokkaido, northern Japan (Fig. S1). The experiments were conducted in the Rawan and Moashoro River basins and in the Toshibetsu and Ashoro River basins as a control. Upland of the Rawan and Moashoro Rivers is a boreal mountain forest (Mt. Meakan, 1499 m altitude), consisting of coniferous trees such as *Picea glehnii*. Agricultural lands are used for pasture and fields (wheat, sugar beet, and adsuki bean, etc.). A manipulation experiment was conducted in an experimental field (43° 17′ 43″ N, 143° 34′ 09″ E) located near the Toshibetsu River (Fig. S1). The annual and growing season (April–June) precipitation amounts in the town of Ashoro from 2010 to 2019 are 842 mm and 210 mm, respectively, and the temperatures are 6.6 °C and 11.0 °C, respectively.

### Stream water quality

Synoptic stream water sampling was conducted in this study area (Fig. S1). Tributary stream water was also collected in the Rawan and Moashoro Rivers. The sampling was conducted every 1–3 months (August 2015, September 2015, October 2015, November 2015, January 2016, April 2016, and July 2016) during the periods with no precipitation from August 2015 to July 2016.

The samples were analyzed for EC, major ions (Cl^−^, NO_3_^−^, SO_4_^2−^, Na^+^, NH_4_^+^, K^+^, Mg^2+^, and Ca^2+^), and SRP. The EC was measured in unfiltered aliquots of stream water. The EC was measured with a conductivity meter (CM-60 V, Toa, Japan). For SRP, aliquots were filtered through precleaned 0.7 µm membrane filters (GF/F, Whatman, GE Healthcare, UK). We then measured the SRP contents by using molybdenum blue (ascorbic acid) absorptiometry (Shimadzu UV mini-1240, Japan). Filtered samples were also passed through a 0.45 µm membrane filter (GL Science, Chromatodisc, 25A) to quantify the major ions. Major ions were analyzed by using an ion chromatograph (DX-120, Dionex, Japan).

### Growth and environmental conditions of Rawanbuki

The growth of Rawanbuki, soil physicochemical properties and stream water quality close to each site were evaluated at three sites along the Rawan River (R1, R2, and R3) and at three sites along the tributary of the Toshibetsu River (T1, T2, and T3) as control sites (Fig. S1). At each site, a 2 m × 2 m plot was set up, and butterburs were harvested in these plots on 21–22 June 2016 when most butterburs were fully grown. After harvesting the butterburs, stem height, stem diameter, and leaf width were measured. The stem height and leaf width were measured with a tape measure, and the stem diameter was measured with a caliper. The harvested samples were dried at 80 °C for 72 h using oven (DKN-812, Yamato, Japan), and the mass of the leaves and stems was determined.

Soil physicochemical properties, such as water content, temperature, pH (H_2_O), EC, inorganic N pool, net N mineralization/nitrification rate, and available P pool, were measured. Soil water content and temperature at a depth of 5 cm were measured in situ at five points in the plot. Surface soil samples at depths of 0–5 cm were collected at five points in the plot to measure chemical properties. Five samples were collected within each site.

Following collection, soils were transported to the laboratory and sieved through a 2-mm mesh sieve to remove coarse fragments. To measure the inorganic N pool^32^, 3 g of the sieved subsamples were immediately shaken separately with 50 mL of 2 M KCl for 1 h to extract NH_4_^+^-N and NO_3_^−^-N. The extract solution was analyzed for NH_4_^+^-N and NO_3_^−^-N for each sample. The NH_4_^+^-N concentration was determined by the indophenol blue method. The NO_3_^−^-N concentration was determined spectrophotometrically after cadmium reduction.

To measure net N mineralization/nitrification rates^32^, the sieved subsamples were incubated in an incubator at 20 °C for 2 months. During the incubation, the amount of distilled water equal to the decrease in the water in the incubated soil was added into the incubated samples every 10 days to maintain soil water contents. To minimize the soil water loss and to ventilate, parafilm with partially opened holes was wrapped around the soil container. Net nitrogen mineralization^32^ (mg N kg^−1^ d^−1^) was calculated as the difference in the concentration of soil NO_3_^−^-N + NH_4_^+^-N between the incubated and initial samples. Net nitrification^32^ (mg N kg^−1^ day^−1^) was also calculated as the difference in the concentration of soil NO_3_^−^-N between the incubated and initial samples. All N data were presented on an oven-dried-weight basis.

Soil pH (H_2_O), EC, and available P pool were measured in the sieved dry soils. The sieved soil was air dried for 10 days. Soil pH was measured in H_2_O (a dry soil:distilled water ratio of 1:2.5) using a glass electrode (F-21; Horiba, Kyoto, Japan), and the EC was measured in H_2_O (a dry soil:distilled water ratio of 1:5) using a conductivity meter (CM-60 V, DKK-TOA Corp., Japan). Available P was determined colorimetrically by the ammonium molybdate method following extraction by the Bray II method^33^.

The N and P concentrations of the leaves and stems of the harvested butterburs were measured to determine the nutrient status of the butterburs. The total N content in the leaves and stems of the butterburs was measured using a CN analyzer (CN recorder MT-700, Yanaco Co., Ltd., Tokyo, Japan). To analyze total P^32^, the dried samples were baked at 550 °C for 2 h and then digested using potassium peroxydisulfate (K_2_S_2_O_8_). The total P concentration in the digested solution was measured using the molybdenum blue (ascorbic acid) spectrophotometric method (UV mini-1240, Shimadzu, Kyoto, Japan). To ensure an accuracy within 5% of known N and P concentrations, a standard reference material (NIST 1515 apple leaves, National Institute of Standards and Technology, Maryland, USA) was analyzed in addition to the butterbur samples.

### Manipulation experiment

Four different treatment plots [control (C), water (W), nutrient (N), and nutrient + water (N + W)] were established (1 m width, 4 m length, and 0.5 m deep). Before the experiments, cut roots of the butterburs (10–15 cm length), obtained at site T2 on 20 October 2016, were planted in four plots in the experimental field. Each plot was filled with volcanic ash soil collected near site T2. In each plot, 7 cut roots were planted at 25 cm intervals in two rows. The plotted butterburs were grown until the manipulation experiment in 2018.

Treatments were conducted for a growing period of 10 weeks from 23 April 2018 to 29 June 2018 and from 22 April 2019 to 28 June 2019. During the study period, the butterburs started and ended shoot growth. The treatment was applied to surface soil two times per week at 1- to 3-day intervals in 2018 and 2019. Potassium nitrate (KNO_3_), potassium sulfate (K_2_SO_4_), tripotassium phosphate (K_3_PO_4_), sodium chloride (NaCl), magnesium sulfate (MgSO_4_), and calcium sulfate (CaSO_4_) were used as the nutrient treatments. The total doses of N, P, K, Mg, and Ca were 10 g N m^−2^, 1 g P m^−2^, 29 g m^−2^, 10 g m^−2^, and 20 g m^−2^, respectively, to meet the requirements of nutrients for butterbur growth.

A mist solution (2 L to each plot, corresponding to 0.5 mm) containing KNO_3_, K_2_SO_4_, K_3_PO_4_, and NaCl was applied using an electric spray machine with a nozzle. MgSO_4_ and CaSO_4_ were applied as powders. For the control solution, a 1000-fold diluted mist solution of the abovementioned mist solution was applied to plots C and W. Irrigation was applied on surface soil every morning (1.5 h) at a rate of 0.8 mm hr^−1^ (GTA111, Takagi, Japan), and total irrigation was 84 mm, corresponding to approximately 40% of the precipitation depth during the butterbur growing season (April–June) in this region.

After the treatments, the maximum net CO_2_ assimilation rates (*A*_max_) were measured using a portable gas exchange measurement system (LI-6400; LI-COR, Lincoln, Nebraska, USA) at near-saturating irradiance (PPFD; 1500 μmol·m^**–**2^ s^**–**1^) for intact leaves during the morning on 8 July 2019. The CO_2_ concentration of the air entering the leaf chamber was maintained at current ambient air concentration of 400 μmol CO_2_ mol^**–**1^, with a flow rate of 500 μmol s^**–**1^. The leaf temperature in the LI-6400 chamber was maintained at 25 °C to maintain the needle-to-air water vapor deficit at less than 1.1 kPa. On 9 July 2019, all butterburs were harvested, and their stem height, stem diameter, and leaf width were measured. The stem height and leaf width were measured by a tape measure, and the stem diameter was measured by a caliper. The harvested samples were dried at 80 °C for 72 h, and the mass of the leaves and stems was determined. The N concentrations of the leaves of the harvested butterburs were measured to determine the nutrient status of the butterburs. Total N content in leaves was measured using a CN analyzer (CN recorder MT-700, Yanaco Co., Ltd., Tokyo, Japan).

### Data analysis

The Kruskal–Wallis H test was used to determine the differences in butterbur growth conditions and soil physicochemical properties between the Rawan (R1, R2, and R3) and Toshibetsu (T1, T2, and T3) Rivers. Pearson's correlation coefficient (r) was used to examine the relationships between soil physicochemical condition and biomass and stream water nutrient concentration and soil chemical condition. Statistical differences in the growth conditions of the butterburs, net photosynthesis, and leaf N contents between the plots were determined using Tukey’s honest significant difference test followed by analysis of variance. All statistical analyses were carried out using SPSS 22.0J (SPSS Japan Inc.).

## Supplementary Information

Supplementary Information
