# Supplementary material for: Nutrients exported from upland stream water enlarge perennial biomass crops
Source: Sci Rep. 2021 Jan 25;11:2200. doi: 10.1038/s41598-021-81191-x (PMC7835229; doi:10.1038/s41598-021-81191-x)
Supplement: Supplementary file 1 — Supplementary Information [file 41598_2021_81191_MOESM1_ESM.docx]

**Supplementary material**

Nutrients exported from upland stream water in enlarge perennial biomass crops

Masaaki Chiwa^1*^, Yasuhiro Utsumi^1^, Naoaki Tashiro^1^, Yuko Yasuda^1, 2^, Ken'ichi Shinozuka^1, 3^, Yang Ru^1^, Nao Nagano^1^, Shusuke Murata^1^, Takuma Nakamura^1^, Kohei Yamauchi^1^, Yuji Kabemura^1^, Tatsuro Ando^4^, Hiroshi Sawamura^4^

^1^ *Kyushu University Forest, Kyushu University, 394 Tsubakuro, Sasaguri, Fukuoka, 811-2415, Japan*

^2^ *Forestry and Forest Products Research Institute, 1 Matsunosato, Tsukuba, Ibaraki, 305-8687, Japan*

^3^ *Fukuoka Institute of Technology, 3-30-1 Wajiro-higashi, Higashi-ku, Fukuoka, 811-0295 Japan.*

^4^ *Ashoro Museum of Paleontology, 1-29-25 Konan, Ashoro, Hokkaido, 089-3727, Japan*

*Corresponding author: Masaaki Chiwa, tel. +81-156-25-2608, fax +81-156-25-3050, e-mail: mchiwa@forest.kyushu-u.ac.jp

The SI contains nine figures and two tables.

**Figure S1** a) Location of the experimental site, Hokkaido, northern Japan. b) map of the locations of synoptic sampling sites of main stream (solid circles) and tributary (solid triangle) to main stream, the sampling locations of butterbur (open circles), and the locations for manipulation experiment (solid square). Numbers in circles and triangles correspond to sampling sites shown in Table S1. The categorization of land uses into forestry, agriculture and urban was determined using National Land Information Division, National Spatial Planning and Regional Policy Bureau, MLIT of Japan land use data set in 2016 download site (https://nlftp.mlit.go.jp/ksj/gml/datalist/KsjTmplt-L03-b.html). The map was generated from ArcGIS version 10.4.1 (ESRI).

**Figure S2** Characteristics of stream water quality in Rawan, Moashoro, Ashoro, and Toshibetsu Rivers. See Fig. S1 for sampling locations of Rawan A, Moashoro A, Ashoro A, and Toshibetsu A.

** Figure S3** Bedrock geological composition of the watershed in Rawan A, Moashoro A, Ashoro A, and Toshibetsu A. See Fig. S1 for sampling locations of Rawan A, Moashoro A, Ashoro A, and Toshibetsu A. Geological data was obtained from Geological Survey of Japan, AIST Geological Survey data set download site (<https://gbank.gsj.jp/geonavi/geonavi.php>). The categorization of bedrock geology was determined by 1/200,000th scale geology maps provided by Geological Survey of Japan (data set download site: https://gbank.gsj.jp/geonavi/geonavi.php). The proportion of each bedrock geology around each sampling point was calculated from the proportion of each geological area to the watershed area at each sampling point. Watershed area at each sampling site was determined by ArcGIS (version 10.4.1).

**Figure S4** Relationships between soil chemical condition and stream water quality including a) pH, b) EC, c) phosphorus, and d) nitrogen.

**Figure S5** Butterbur tissue N concentration of a) leaf and b) stem; tissue P concentration of c) leaf and d) stem. Bars represent standard error (*n* = 5). There were no significant differences between Rawan and Toshibetsu for leaf N, stem N, leaf P, and stem P, *P* = 0.86, 0.09, 0.58, and 0.80, respectively.

**Figure S6** Relationships between a) initial soil NO_3_^-^ and NH_4_^+^ contents (mgN kg^-1^) and above ground N of stem and leaf (mgN kg^-1^) and b) initial soil P contents (mgP_2_O_5_ kg^-1^) and above ground P of stem and leaf (mgP kg^-1^).

**Figure S7** Area-based above ground biomass (g m^-2^) grown on the condition of control (C), addition of water (W), addition of nutrients (N), and addition of nutrients and water (N + W).

**Figure S8** CO_2_ assimilation rate (*A_max_*) of leaf grown on the condition of control (C), addition of water (W), addition of nutrients (N), and addition of nutrients and water (N + W). Bars represent standard errors (*n* = 8).

**Figure S9** Leaf N concentration (mg g^-1^) grown on the condition of control (C), addition of water (W), addition of nutrients (N), and addition of nutrients and water (N + W). Bars represent standard errors (*n* = 8). Different letters indicate significant differences at *P* < 0.05 (Tukey’s honest significant difference test) among treatments.

| Table S1. Stream water quality in this study and other Japanese rivers (μmol L^−1^). | | | | | | | |
| --- | --- | --- | --- | --- | --- | --- | --- |
| River |  | NO_3_^−^ | SRP | Na^+^ | K^+^ | Mg^2+^ | Ca^2+^ |
| Rawan | Main 1 | 19 | 0.06 | 981 | 124 | 640 | 899 |
|  | Main 2 | 11 | 0.46 | 962 | 110 | 403 | 477 |
|  | Main 3 | 166 | 1.57 | 3276 | 451 | 2333 | 1531 |
|  | Main 4 | 151 | 1.19 | 2968 | 409 | 2116 | 1459 |
|  | Main 5 | 140 | 1.13 | 2625 | 370 | 1835 | 1275 |
|  | Main 6 | 133 | 1.27 | 2515 | 337 | 1721 | 1193 |
|  | Main 7 | 81 | 1.02 | 1706 | 208 | 1033 | 790 |
|  | Main 8 | 75 | 0.97 | 1517 | 177 | 852 | 672 |
|  | Tributary 1 | 46 | 0.04 | 240 | 9 | 129 | 312 |
|  | Tributary 2 | 37 | 1.51 | 619 | 52 | 34 | 95 |
|  | Tributary 3 | 51 | 1.54 | 570 | 64 | 43 | 133 |
|  |  |  |  |  |  |  |  |
| Moashoro | Main 1 | 248 | 1.08 | 3856 | 466 | 3076 | 3533 |
|  | Main 2 | 139 | 0.78 | 2591 | 311 | 2055 | 2515 |
|  | Main 3 | 106 | 1.26 | 1746 | 213 | 1231 | 1539 |
|  | Main 4 | 95 | 1.73 | 1521 | 181 | 961 | 1195 |
|  | Main 5 | 91 | 1.66 | 1378 | 166 | 834 | 1041 |
|  | Tributary 1 | 23 | 0.47 | 2119 | 207 | 1805 | 2929 |
|  | Tributary 2 | 16 | 0.96 | 334 | 65 | 136 | 283 |
|  | Tributary 3 | 16 | 0.86 | 558 | 36 | 60 | 144 |
|  | Tributary 4 | 46 | 2.19 | 691 | 44 | 48 | 147 |
|  | Tributary 5 | 22 | 1.27 | 436 | 70 | 82 | 244 |
|  |  |  |  |  |  |  |  |
| Ashoro | Main 1 | 21 | 0.29 | 742 | 83 | 474 | 994 |
|  | Main 2 | 21 | 0.34 | 647 | 77 | 420 | 867 |
|  | Main 3 | 23 | 0.43 | 666 | 85 | 416 | 846 |
|  | Main 4 | 35 | 0.49 | 571 | 77 | 319 | 690 |
|  |  |  |  |  |  |  |  |
| Toshibetsu | Main 1 | 37 | 0.67 | 233 | 47 | 79 | 172 |
|  |  |  |  |  |  |  |  |
| Other Rivers^25^ |  | 17  (0.7−260) | 0.7  (0−7.4) | 323  (91−3401) | 31  (9−133) | 80  (12−568) | 229  (65−893) |
| Number in parenthesis indicate minimum-maximum. | | | | | | | |

| Table S2. Soil physicochemical properties at the investigated sites along the Rawan River and Toshibetsu River. | | | | | | | | |
| --- | --- | --- | --- | --- | --- | --- | --- | --- |
|  | WC  (%) | Temp  (ºC) | pH (H_2_O) | EC  (mS m^−1^) | Initial NO_3_^−^  (mgN kg^−1^) | Net-amm^a^  (mgN kg^−1^ d^−1^) | Net-nit^b^  (mgN kg^−1^ d^−1^) | P contents  (mgP_2_O_5_ kg^−1^) |
| R1 | 54.1 | 13.5 | 6.58 | 29.1 | 49.2 | -0.44 | 2.64 | 248.2 |
| R2 | 52.1 | 13.8 | 6.96 | 19.7 | 15.0 | -0.61 | 4.66 | 160.2 |
| R3 | 55.1 | 13.5 | 6.82 | 15.5 | 14.0 | -0.41 | 2.09 | 216.1 |
| T1 | 45.9 | 12.8 | 5.87 | 7.0 | 2.8 | -0.60 | 2.62 | 29.3 |
| T2 | 49.0 | 15.5 | 5.83 | 5.9 | 1.2 | -0.47 | 1.31 | 23.8 |
| T3 | 52.1 | 13.5 | 6.25 | 8.0 | 1.9 | -0.56 | 2.84 | 48.1 |
| a ammonification; b nitrification | | | | | | | | |

| Table S3. Stream water quality at the investigated sites along the Rawan River and Toshibetsu River (μmol L^−1^). | | | | | | |
| --- | --- | --- | --- | --- | --- | --- |
| Site | NO_3_^−^ | SRP | Na^+^ | K^+^ | Mg^2+^ | Ca^2+^ |
| R1 | 49.2 | 49.2 | 49.2 | 49.2 | 49.2 | 49.2 |
| R2 | 15.0 | 15.0 | 15.0 | 15.0 | 15.0 | 15.0 |
| R3 | 14.0 | 14.0 | 14.0 | 14.0 | 14.0 | 14.0 |
| T1 | 2.8 | 2.8 | 2.8 | 2.8 | 2.8 | 2.8 |
| T2 | 1.2 | 1.2 | 1.2 | 1.2 | 1.2 | 1.2 |
| T3 | 1.9 | 1.9 | 1.9 | 1.9 | 1.9 | 1.9 |
|  | | | | | | |
